# Perturbation of cytokinin and ethylene-signalling pathways explain the strong rooting phenotype exhibited by Arabidopsis expressing the *Schizosaccharomyces pombe *mitotic inducer, *cdc25*

**DOI:** 10.1186/1471-2229-12-45

**Published:** 2012-03-27

**Authors:** Natasha D Spadafora, David Parfitt, Angela Marchbank, Sherong Li, Leonardo Bruno, Rhys Vaughan, Jeroen Nieuwland, Vicky Buchanan-Wollaston, Robert J Herbert, Maria Beatrice Bitonti, John Doonan, Diego Albani, Els Prinsen, Dennis Francis, Hilary J Rogers

**Affiliations:** 1 School of Biosciences, Cardiff University, Cardiff CF10 3AT, UK; 2 Dipartimento di Ecologia, Università della Calabria, Arcavacata di Rende, Cosenza I-87030, Italy; 3 School of Life Sciences, University of Warwick, Wellesbourne, Warwick CV35 9EF, UK; 4 Institute of Science and the Environment, University of Worcester, Henwick Grove, Worcester WR2 6AJ, UK; 5 Plant Phenomics Centre, Institute of Biological, Environmental and Rural Sciences, Penglais, Aberystwyth University, Ceredigion SY23 3DA, Aberystwyth, UK; 6 Department of Botanical, Ecological and Geological Sciences, University of Sassari, Via Piandanna 4, Sassari 07100, Italy; 7 Department of Biology, University of Antwerp, Groenenborgerlaan 171, Antwerp B-2020, Belgium

## Abstract

**Background:**

Entry into mitosis is regulated by cyclin dependent kinases that in turn are phosphoregulated. In most eukaryotes, phosphoregulation is through WEE1 kinase and CDC25 phosphatase. In higher plants a homologous CDC25 gene is unconfirmed and hence the mitotic inducer *Schizosaccharomyces pombe *(*Sp*) *cdc25 *has been used as a tool in transgenic plants to probe cell cycle function. Expression of *Spcdc25 *in tobacco BY-2 cells accelerates entry into mitosis and depletes cytokinins; in whole plants it stimulates lateral root production. Here we show, for the first time, that alterations to cytokinin and ethylene signaling explain the rooting phenotype elicited by *Spcdc25 *expression in Arabidopsis.

**Results:**

Expressing *Spcdc25 *in Arabidopsis results in increased formation of lateral and adventitious roots, a reduction of primary root width and more isodiametric cells in the root apical meristem (RAM) compared with wild type. Furthermore it stimulates root morphogenesis from hypocotyls when cultured on two way grids of increasing auxin and cytokinin concentrations. Microarray analysis of seedling roots expressing *Spcdc25 *reveals that expression of 167 genes is changed by > 2-fold. As well as genes related to stress responses and defence, these include 19 genes related to transcriptional regulation and signaling. Amongst these was the up-regulation of genes associated with ethylene synthesis and signaling. Seedlings expressing *Spcdc25 *produced 2-fold more ethylene than WT and exhibited a significant reduction in hypocotyl length both in darkness or when exposed to 10 ppm ethylene. Furthermore in *Spcdc25 *expressing plants, the cytokinin receptor *AHK3 *was down-regulated, and endogenous levels of iPA were reduced whereas endogeous IAA concentrations in the roots increased.

**Conclusions:**

We suggest that the reduction in root width and change to a more isodiametric cell phenotype in the RAM in *Spcdc25 *expressing plants is a response to ethylene over-production. The increased rooting phenotype in *Spcdc25 *expressing plants is due to an increase in the ratio of endogenous auxin to cytokinin that is known to stimulate an increased rate of lateral root production. Overall, our data reveal important cross talk between cell division and plant growth regulators leading to developmental changes.

## Background

Proliferative cells are made competent for DNA replication and mitosis at the G1/S and G2/M boundaries. In Arabidopsis, at G2/M, proliferative cells are regulated by Arath;CDKA;1, and by B-type CDKs, one of which, CDKB1;1, has a single peak of activity at G2/M [1]. In fission yeast, at the G2/M transition, Mik1/Wee1 kinases act redundantly to phosphorylate Tyr15 of the CDK thereby inactivating the latter [2]. Conversely Cdc25 dephosphorylates the same residue enabling CDK activity [3]. Although homologues for the fission yeast *wee1 *have been identified in several plant species [4-6], a full length homologue of *CDC25 *has only been found in algae [7] while higher plant genomes have a partial CDC25 gene that lacks the regulatory domain [8,9]. Although *Arath;CDC25 *(At5g03455) encodes a protein capable of phosphatase activity *in vitro *[8], it induces a short cell length in fission yeast [9] and has a subtle effect on root growth [10], its encoded protein can exhibit arsenate reductase activity [11,12]. Thus currently, there is insufficient functional evidence to tag *Arath;CDC25 *as a *bona fide *CDC25 cell cycle gene. However, in *Nicotiana plumbaginifolia *cell cultures a cdc25-like phosphatase activity was detected at the G2/M transition [13] although the identity of the gene involved remains unknown.

In the absence of a clear plant CDC25 homologue, *Schizosaccharomyces pombe Spcdc25 *expression in plant cells has been useful as a tool to investigate the effects of dephosphorylation of the CDKs on cell division and plant development. *Spcdc25 *has clear effects on development and on the cell cycle when expressed in tobacco. In *N. plumbaginifolia *it dephosphorylated native CDK and induced cells to enter mitosis [14]. It also induced a small cell size in tobacco plants [15], in tobacco root cultures [16] and in tobacco BY-2 cells [17]. In BY-2 cells, the smaller mitotic cell size induced by *Spcdc25 *expression was linked to CDKB but not CDKA activity, and a short G2 phase [17]. Furthermore, in cultured primary roots of tobacco, induced *Spcdc25 *expression caused an increase in the frequency of smaller lateral root primordia, and smaller roots comprising smaller mitotic cells compared with un-induced roots [16].

Expression of *Spcdc25 *in tobacco cell cultures suggested a link to cytokinin signalling. In plants, cytokinins are required for the G2/M transition [18,19] and treating tobacco BY-2 cells with lovastatin, an inhibitor of cytokinin biosynthesis, not only suppressed a peak of cytokinin synthesis but also blocked the G2/M transition [18]. However expression of *Spcdc25 *in tobacco BY-2 cells could over-ride this requirement [14,17] suggesting that the additional phosphatase activity was replacing the cytokinin signal. Furthermore, all moieties of cytokinin were barely above detectable levels in *Spcdc25 *expressing cells [17]. In tobacco plants expressing *Spcdc25*, induction of both vegetative and floral shoot morphogenesis from callus suggests that the *Spcdc25 *expression was mimicking an altered balance of plant growth regulators towards cytokinins; *Spcdc25 *could replace exogenous cytokinin to induce shoots in culture [20,21]. However, to date, evidence of alterations to endogenous cytokinins that could strengthen the cell cycle link of *Spcdc25 *to developmental changes is lacking in whole plants.

A two-hybrid screen revealed that Spcdc25 interacts with numerous Arabidopsis proteins including 14-3-3 proteins [22], however to date there is no information on changes in endogenous gene expression as a result of the expression of *Spcdc25 *in plants. A major aim of the work presented here was to test the relationship between *Spcdc25 *expression and endogenous cytokinins and to analyse global gene expression in Arabidopsis plants expressing *Spcdc25*, in which we have also made a detailed phenotypic analysis.

Results presented here extend substantially our previous work in showing that ectopic expression of the mitotic inducer, *Spcdc25*, results in profound changes to root morphology, meristem architecture and the regulation of the plane of cell division. We further show that *in planta Spcdc25 *expression (1) depletes endogenous levels of the cytokinin, iPA, (2) down regulates the cytokinin receptor, *AHK3*, (3) results in over production of auxin and ethylene, (4) up-regulates ethylene biosynthetic genes and (5) induces hypersensitive hypocotyl growth responses to ethylene. Hence perturbation of genes associated with cytokinin and ethylene signalling explain the phenotypes upon expression of this fission yeast mitotic inducer gene. This indicates an important cross-talk between cell cycle regulation at G2/M transition, plant growth regulators and plant morphogenesis.

## Results

### Expression of *Spcdc25 *increases the frequency of lateral roots and adventitious roots but not primary root elongation in Arabidopsis seedlings

**Figure 1:**
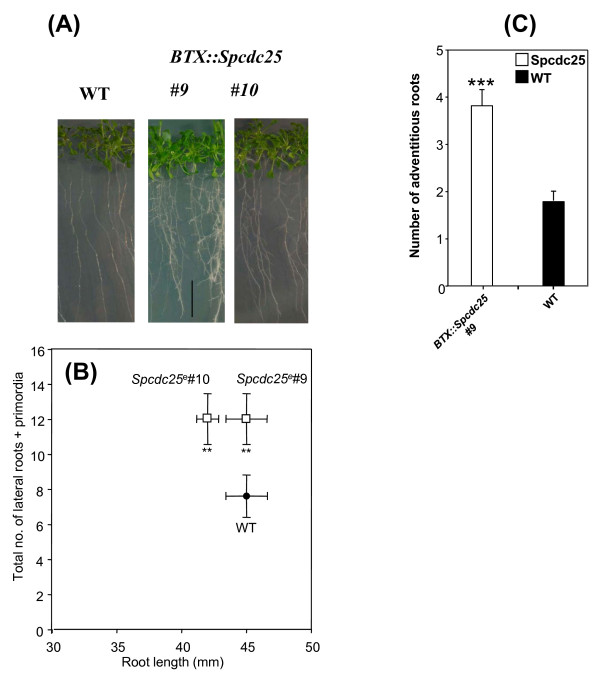
***Spcdc25 *expression stimulates lateral root growth**. (A) Phenotypes of 10 day-old Arabidopsis seedlings: Wild type (WT), and BTX::*Spcdc25 *(lines 9 and 10). Bar scale = 10 mm. (B) The relationship between mean total number of lateral roots + lateral root primordia (± SE) and mean primary root length (mm ± SE) for 10 day old seedlings grown at 21°C: Wild type (WT), and two *Spcdc25 *expressing lines, 9 and 10. Root length in Spcdc25 lines 9 and 10 was not significantly different from WT (P > 0.05). Number of laterals + primordia were significantly different from WT (p = **0.02) in Spcdc25 lines 9 and 10 cf WT. The rate of lateral root formation mm primary root^-1 ^for each genotype: WT = 0.17, Spcdc25 line 10 = 0.28, Spcdc25 line 9 = 0.26. (C) Adventitious root phenotypes in WT and BTX::Spcdc25 line 9, in 24 d old seedlings. All data are means ± SE; levels of significance (P) are indicated by Student's *t*-test: P *** < 0.001, P ** 0.02-0.001 P* 0.02-0.05; n = 25

Given the root developmental changes induced by *Spcdc25 *in tobacco, we tested whether *Spcdc25 *could similarly affect Arabidopsis. Two independent transgenic lines expressing the *BTX:: Spcdc25 *construct, verified by RT-PCR (shown in Additional file 1), showed a significant increase in the number of lateral roots and lateral root primordia per unit length of primary root, compared with WT in 10 d old seedlings but no difference in primary root length. (Figure 1A and 1B). Hence, the rate of lateral root production per mm of primary root was substantially higher (1.6-fold) in the Spcdc25 lines compared with WT (Figure 1).

We also tested whether expression of *Spcdc25 *affected the formation of adventitious roots from cultured hypocotyls derived from intact 24 d old plants and found that Spcdc25 explants produced significantly more than WT (Figure 1C).

### Expression of *Spcdc25 *decreases root meristem width through narrowing of cortical and stelar cell width

**Figure 2:**
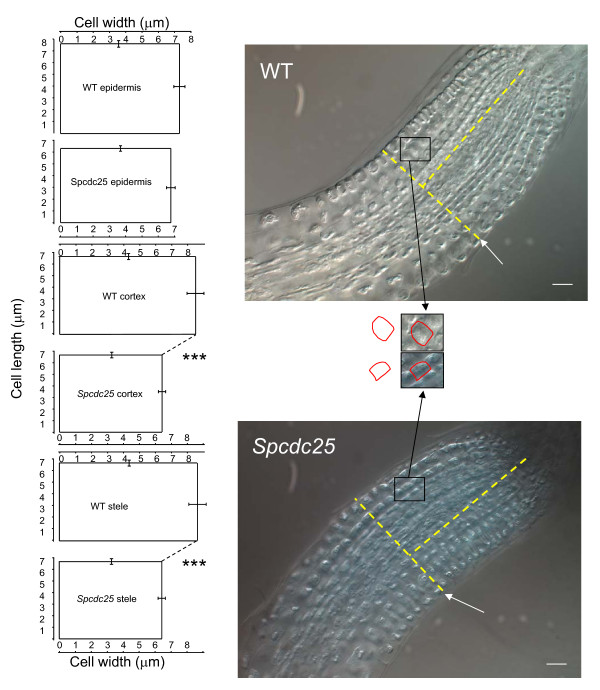
**Narrower cell width and root width in *Spcdc25 *RAMs**. The left-hand panel comprises mean (± S.E) cell length and width (μm) in epidermal, cortical and stelar lineages extending from the tip of the RAM to the transition point for each tissue for the wild type (WT) and *Spcdc25 *line *9 *expressing genotypes (levels of significance (P) are indicated by Student's *t*-test: *** P < 0.001; n = 50). The right hand panel comprises representative 10-day-old WT- and *Spcdc25 *expressing-whole root tips imaged by DIC/Nomarski microscopy. Solid white arrows indicate the Transition Point for the epidermal lineage. The overlaid T junctions at the transition point enable a measurement of root width at comparable distances from the apex of the RAM for both genotypes. Blow ups were then made for a cortical cell from both genotypes to show more clearly the reduction in cell size in *Spcdc25 *cf. WT. scale bars = 20 μm.

A hall-mark feature of *Spcdc25 *expression in tobacco plants is a small cell size phenotype and we examined whether this was also so in Arabidopsis. We extended this analysis in Arabidopsis to examine RAM architecture in relation to the similar rate of primary root elongation of Spcdc25 compared with WT. *Spcdc25 *expression affected neither cell length nor cell width for epidermal lineages of the RAM. Indeed, *Spcdc25 *expression also had little effect on cell length in either the cortex or stele (Figure 2). However, there was a highly significant decrease in cell width for cells of the cortex and stele in Spcdc25 compared with WT (Figure 2). Given little change in cell length between genotypes, the transition point was at comparable distances from the root tip for each tissue (e.g. epidermis in Figure 2). However, consistent with the narrowing of cell width in the cortex and stele, the overall girth of the RAM was narrower (P < 0.01) in Spcdc25 (91.67 ± 1.90 μm) compared with WT (124 ± 3.70 μm) (Figure 2).

**Figure 3:**
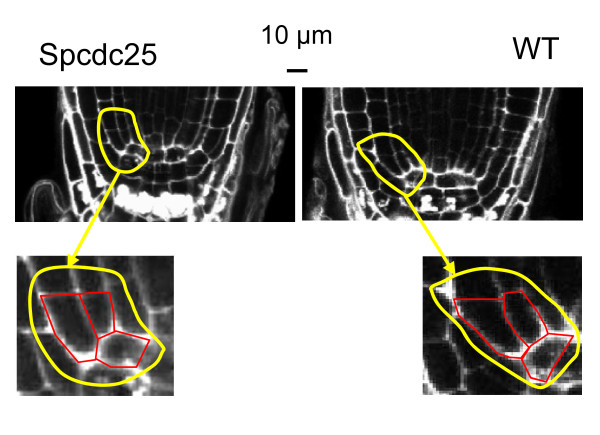
***Spcdc25 *RAM has narrower cells in Körper ⊥ divisions compared with wild type**. Median confocal images of promeristems of *Spcdc25 *and WT, each with a tracing around a Körper division immediately adjacent to the quiescent centre, and a subsequent "blow up" of this cluster of cells. Bar scale = 10 μm.

At the cellular level, the data suggest that in the cortex and stele, *Spcdc25 *expression reduces the span of periclinal divisions that in turn would create narrower cell lineages. We tested this hypothesis using confocal microscopy. Here we show that *Spcdc25 *expression does indeed result in narrower "Körper clusters" compared with wild type (Figure 3 and see Discussion). The net result is a narrower RAM and a narrower primary root system compared with WT. However, clearly, *Spcdc25 *expression has no effect on anticlinal divisions in the epidermis. The null effect of *Spcdc25 *on meristematic cell length is consistent with no significant difference in meristem length between genotypes (data not shown) and similar rates of primary root elongation between genotypes (Figure 1). Consistent with this is that in cortical tissue adjacent to young lateral root primordia, mean cortical cell lengths of 75 ± 10.1 and 56.86 ± 10.3 μm for WT and *Spcdc25*, respectively were not significantly different (n = 25, see Additional file 2).

### Expression of *Spcdc25 *increases rooting from cultured hypocotyls

In tobacco cell cultures expression of *Spcdc25 *enables cells to bypass a requirement for cytokinins at the G2/M transition; indeed in this line cytokinin levels were depleted [17]. The strong lateral root phenotype *in vivo *also suggested that auxin levels might be involved in the plant's response to *Spcdc25 *expression. To explore further this cross-talk between plant growth regulators and the cell cycle, we tested whether application of exogenous cytokinin and auxin might affect rooting in cultured hypocotyls.

In initial experiments we used the grid system of Inoue *et al*. [23] comprising exposure of explanted hypocotyls to combinations of naphthyl acetic acid (NAA) and Kinetin (Kin) ranging from 25 to 300 ng ml^-1 ^(shown in Additional file 3). From here on we refer to these treatments as NAA/Kin, each prefixed by the appropriate concentration (ng ml^-1^).

At higher levels of NAA/Kin (e.g. ≥ 200NAA) callus and root/shoot formation appeared similar in *Spcdc25 *and WT, however at 100 NAA, root/shoot formation appeared enhanced in WT compared with *Spcdc25*. Conversely at ≤ 50 NAA, root formation appeared enhanced in *Spcdc25 *compared with WT (NAA/Kin: 50/25, 50/200, 25/100, 25/200). Hence, there was the suggestion, that below an auxin threshold (≤ 50 NAA), hypocotyls from Spcdc25 plants explanted to culture media have increased competence for root formation.

**Figure 4:**
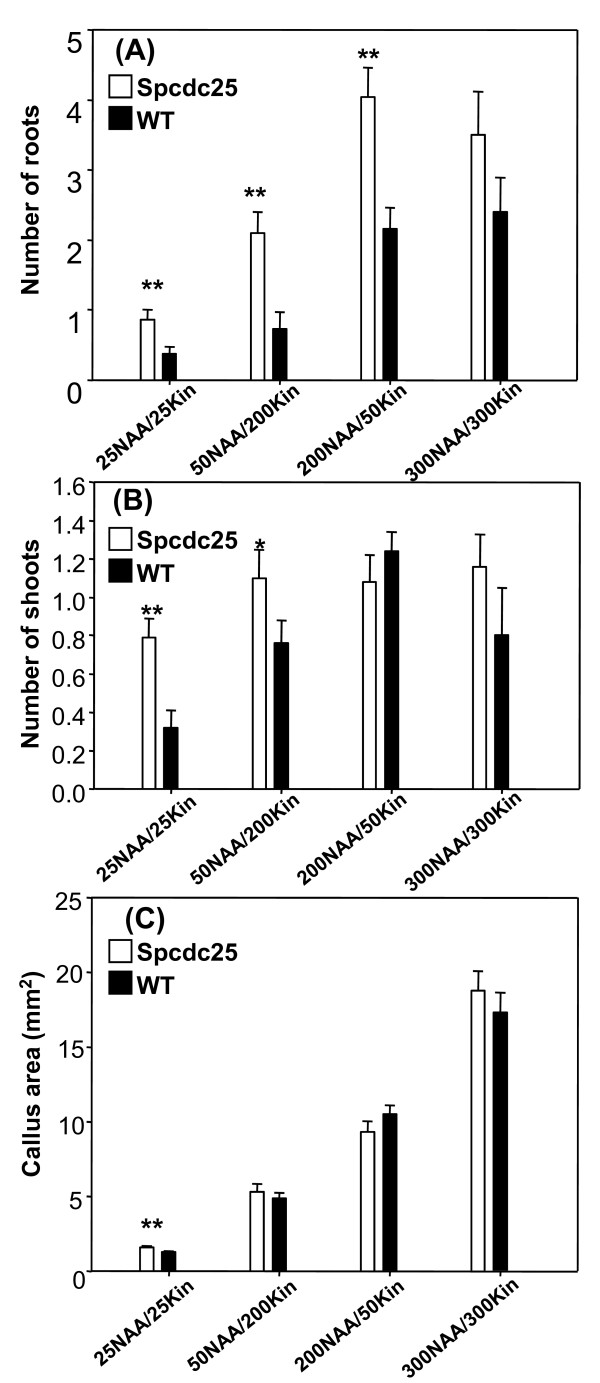
**Spcdc25 induces stronger rooting response at ≤ 50NAA**. Mean (± S.E.) number of (A) roots (B) shoots and (C) callus area in cultures of 14 day old hypocotyls from *Spcdc25 *expressing line 9 (open bars), and wild type (solid bars) cultured on MS medium supplied with a range of concentrations of kinetin (Kin ng ml^-1^) and naphthyl acetic acid (NAA ng ml^-1^) for 30 d. Significance levels are P values from Student t-tests between the transgenic line and WT: *0.05-0.02, ** 0.02-0.001. n = 25

To verify these indicative results we undertook a more rigorous quantitative analysis of this trend by employing four specific NAA/Kin combinations in which we established 25 hypocotyls per treatment (Figure 4). We chose 25NAA/25Kin and 300NAA/300Kin, the minimum and maximum concentrations used in the grids. We further chose 50NAA/200Kin as a combination that induced a stronger rooting response in Spcdc25 compared with WT in the original grids and the converse, 200NAA/50Kin. At all of the NAA/Kin combinations tested except the highest, there were significantly more roots produced by the transgenic line expressing *Spcdc25 *compared to WT (Figure 4A) whereas shoot production was significantly higher only at the two lower auxin concentrations (Figure 4B). Callus formation was only significantly greater in the *Spcdc25 *expressing line at the lowest auxin and cytokinin concentrations tested (Figure 4C).

### Expression of *Spcdc25 *in Arabidopsis affects expression of 167 genes including genes related to ethylene signalling

To understand more fully how the expression of *Spcdc25 *caused clear phenotypic changes in Arabidopsis roots we examined the extent to which the expression of this gene caused alterations in global gene expression occurring in the transformed seedlings. Microarray analysis of Arabidopsis seedling roots expressing *Spcdc25 *compared to WT revealed that 87 genes were up-regulated > 2-fold and 80 were down-regulated by > 2-fold when analysed using Genespring software (see Additional file 4 for the full list of genes). Expression of selected genes was confirmed by real time RT-PCR, (see Additional file 5 for the real-time PCR data).

Of particular interest were 20 differentially expressed genes related to signalling, transcription and plant growth regulators (Table 1), 10 of which were up-regulated while 10 were down regulated in the *Spcdc25 *line. Three of the differentially expressed genes are related to ethylene signalling. The first (At5g61590), which is a member of a subfamily B-3 of the ethylene responsive *ERF/AP2 *transcription factors, is up-regulated in *Spcdc25 *seedlings by 2.6-fold. The other two genes are down regulated by 2.0-fold and 5.0-fold respectively: At2g27050, which is a member of the EIN3 family, positive regulators of ethylene responses and At2g25490, known as *EBF1*, an F-box protein involved in the ubiquitin/proteasome-dependent proteolysis of EIN3, and is thus a negative regulator of ethylene signalling [24]. In addition *ACC OXIDASE *(At1g12010) expression was up-regulated on the microarrays by 2.6-fold.

**Table 1:** genes with putative functions in transcriptional regulation or signalling whose expression changed by > 2-fold in roots of Arabidopsis seedlings expressing Spcdc25 compared to WT

Ratio Spcdc25/WT	*t*-test P-value	ATG code	Putative function
2.6	0.009	At5g61590	member of the ERF (ethylene response factor) subfamily B-3 of ERF/AP2 transcription factor family

2.6	0.002	At1g12010	1-aminocyclopropane-1-carboxylate oxidase putative/acc oxidase putative

2.3	0.001	At5g59780	regulation of transcription, response to ethylene gibberellin, jasmonic acid, and salicylic acid stimuli

2.2	0.001	At5g20030	RNA binding protein

2.1	0.019	At5g54930	AT hook motif-containing protein; functions in: DNA binding

2.1	0.036	At1g48260	SNF1-related kinase (SnRK) member of the CBL-interacting protein kinases (CIPK17).

2.1	0.045	At5g53450	OBP3-responsive gene 1 (ORG1); functions in: protein kinase activity, kinase activity,

2.0	0.040	At1g69220	serine/threonine kinase (SIK1) similar to yeast gene PAK1 involved in cytokinesis, actin polarization.

2.0	0.042	At1g08320	bZIP family transcription factor

2.0	0.004	At3g56970	OBP3-RESPONSIVE GENE 2, member of the basic helix-loop-helix transcription factor family

0.2	0.000	At2g25490	Negative regulation of ethylene signalling F-box protein involved in the proteolysis of EIN3

0.3	0.002	At5g42020	Luminal binding protein (BiP2) involved proliferation of endosperm nuclei, response to stress

0.3	0.029	At5g14580	polyribonucleotide nucleotidyltransferase, involved in RNA processing, stability

0.4	0.034	At4g24190	SHD: ER-resident HSP90-like protein and is involved in regulation of meristem size and organization

0.4	0.020	At1g27320	AHK3: histidine kinase, a cytokinin receptor.

0.5	0.001	At1g20823	zinc finger (C3HC4-type RING finger) family protein, response to chitin, a plant-defense elicitor.

0.5	0.031	At2g27050	transcription factor, ethylene-insensitive3-like1 (EIL1), response to ethylene stimulus

0.5	0.044	At2g02170	remorin family protein; functions in: DNA binding, high expression in SAM

0.5	0.009	At1g49760	polyadenylate-binding protein, putative/PABP, putative, similar to poly(A)-binding protein,

0.5	0.019	At2g01150	RING-H2 finger protein ubiquitin-protein ligase activity

### Spcdc25 expression results in changes linked to ethylene metabolism and signalling

**Figure 5:**
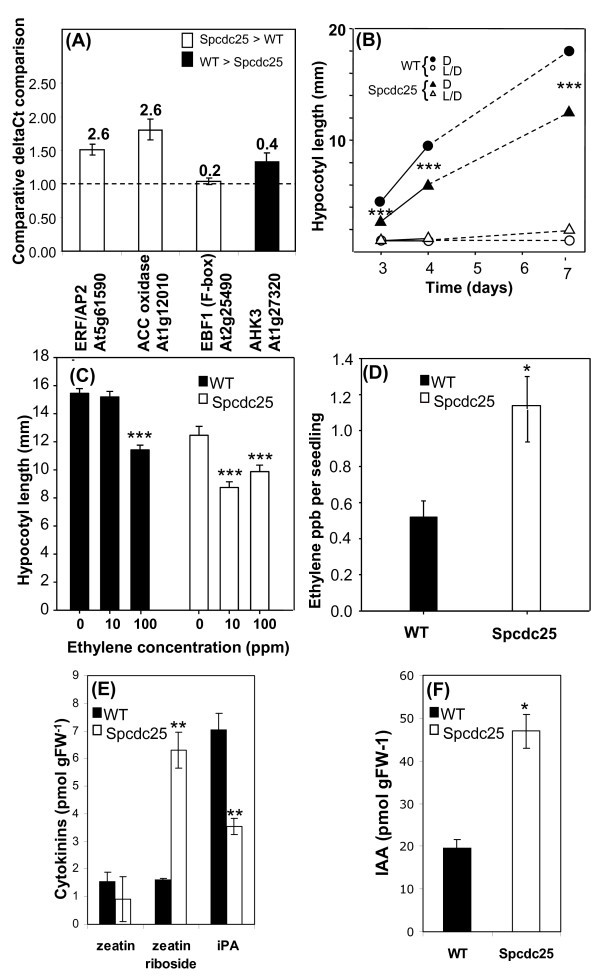
**Spcdc25 alters ethylene, cytokinin and auxin synthesis, expression of ethylene and cytokinin signalling genes and hypoctyl growth responses**. (A) real time PCR analysis of relative expression of four genes related to ethylene and cytokinin signalling in roots of 10 day old seedlings. n = 3. (Above each histogram bar is the array result Spcdc25/WT); (B) relationship between hypocotyl length (mm) and time (days), in seedlings grown in dark (D, closed symbols) or 16 h L, 8 h D (L/D, open symbols), (error bars < diameter of symbols) n = 25; (C) hypocotyl length in seedlings exposed to 0, 10 or 100 ppm ethylene for 10 days. n = 20; (D) endogenous ethylene levels (ppb per seedling) in 10 day old seedlings in WT and Spcdc25 n = 3; number of seedlings measured in each replicate sample, WT 1. 273, 2. 238, 3. 413; Spcdc25 1. 181, 2. 276, 3. 188. (E) endogenous cytokinins and (F) endogenous IAA, in whole root systems of 10 day old seedlings. n = 3. C-F: WT (black bars), Spcdc25 (white bars). All data are means ± S.E levels of significance (P) are indicated by Student's *t*-test: P *** < 0.001, P ** 0.02-0.001 P* 0.02-0.05

The microarray results provided novel information about genes associated with ethylene metabolism in the Spcdc25 plants. Expression levels of the ethylene related genes *ERF2/AP2 *family member (At5g61590), *ACC OXIDASE *(At1g12010) and *EBF1 *(At2g25490) were therefore verified using real time RT-PCR. Expression of both *ERF2/AP2 *and *ACC OXIDASE *were up-regulated by at least 1.5-fold in agreement with the array data. The down-regulation of *EBF1 *on the array, however was not confirmed (Figure 5A). These data are consistent with increased ethylene production and signalling in Spcdc25 plants.

Given the development of isodiametric cells in the cortex and stele of RAMs in *Spcdc25 *expressing plants, which is a typical ethylene induced response [25], we tested for further evidence of ethylene-related phenotypes in the Spcdc25 plants. Hypocotyl length was highly significantly reduced (P < 0.001) in 3, 4 and 7 day old seedlings of Spcdc25 plants compared with WT grown in continuous darkness (Figure 5B). This differential effect was nullified when the seedlings were grown in a 16 h L/8 h D regime. Exposing these genotypes to exogenous ethylene also resulted in a differential magnitude of hypocotyl shortening. In WT, hypocotyl length was not significantly different in the 0 and 10 ppm ethylene treatments but there was a highly significant difference at 100 compared with 0 ppm (Figure 5C). In contrast, in Spcdc25 hypocotyl length was significantly shorter in the 10 ppm treatment compared with 0 ppm but there was no additional shortening at 100 ppm although compared with 0 ppm, hypocotyl length was significantly shorter (Figure 5C). These data suggest a hypersensitive response of hypocotyls of Spcdc25 plants compared with WT when exposed to 10 ppm ethylene. Moreover, Spcdc25 plants produced 2-fold more ethylene than WT (Figure 5D).

### *Spcdc25 *expression in Arabidopsis perturbs the expression of a cytokinin receptor gene and perturbs endogenous cytokinin and IAA levels

Notable in the microarrays, was the down-regulation of *AHK3 *in Spcdc25 plants by 2.5-fold; this gene encodes a putative cytokinin receptor. This result was confirmed by real-time RT-PCR (Figure 5A). We then tested whether endogenous cytokinin levels mirrored *AHK3 *expression, in roots of Spcdc25 and WT. Data presented in Figure 5E for roots of Spcdc25 plants compared to WT show a 4.8-fold increase in zeatin riboside (ZR) concentration, zeatin (Z) was unaltered whereas isopentenyladenosine (iPA) was reduced by almost 2-fold (Figure 5E). Hence, the cytokinin depletion effect we had observed in tobacco cells in culture was matched regarding iPA in root systems of Arabidopsis. These changes in cytokinins may help to explain the enhanced lateral root phenotype in Spcdc25 plants. Additionally, we detected a substantial (2-fold) increase in free IAA in Spcdc25 roots (Figure 5F), again consistent with the enhanced root branching response.

## Discussion

### *Spcdc25 *expression promotes lateral and adventitious root production

In tobacco plants, expression of *Spcdc25 *resulted in a decrease in mitotic cell size and an increase in lateral root production [16]. Results here confirm this effect in Arabidopsis but show that whilst primary root elongation is not promoted by *Spcdc25 *expression, lateral and adventitious root production most certainly is.

Although *in vivo *responses are not always mirrored *in vitro *we further showed this strong rooting phenotype *in vitro *through culture of hypocotyls in two way combinations of auxin and cytokinin. Noticeably, at the lower end of the auxin concentration range (≤50 mM NAA) but seemingly regardless of exogenous kinetin levels, explanted hypocotyls from Spcdc25 plants were more adept at making roots *in vitro*. Thus hypocotyls showed greater competence for rooting *in vivo *and *in vitro *confirming cross talk between this mitotic inducer gene and development. Indeed, we propose that the increased frequency of lateral roots is a cell cycle effect in the pericycle, based on published evidence that *Spcdc25 *dephosphorylates native plant CDK [14] and induces both premature CDKB activity and premature mitosis [17].

### *Spcdc25 *expression depletes iPA and raises IAA

One way in which lateral root production can be enhanced is by cytokinin depletion through over-expression of *CYTOKININ OXIDASE *[26]. *Spcdc25 *expression also depletes cytokinin levels in cultured BY-2 cells [17] and led us to hypothesise that a similar *Spcdc25-*induced reduction in cytokinin levels in Arabidopsis roots might explain the root phenotypes we observe. Cytokinin levels in these plants partly support this hypothesis in that iPA is significantly depleted but neither zeatin nor zeatin riboside are reduced. However, in plants expressing *Spcdc25*, the cytokinin receptor gene, *AHK3 *showed reduced expression. Note that there is not a universal positive relationship between cytokinin depletion and root branching. For example, Arabidopsis *ahk2 ahk3 *double mutants had increased zeatin and zeatin riboside levels, in combination with decreased iPA but increased primary root growth as well as more lateral roots [27]. Thus the data presented here, showing a reduction in *AHK3 *expression with a depletion of iPA level in combination with an increase in trans-zeatin riboside is also consistent with an increase in lateral root production in the *Spcdc25 *expressing plants. Riefler et al., [27] argue for a feedback mechanism between receptor levels and cytokinin biosynthesis. An increase in mitotic entry by *Spcdc25 *expression might be feeding back both to cytokinin levels and also to receptor levels with more emphasis on depletion of iPA than other cytokinin moieties.

The steady state level of endogenous cytokinins in plant tissues is a reflection of cytokinin biosynthesis, degradation and transport. Nucleoside-type cytokinins have been proposed as the major transport form [28-31]; iP ribosides and ribotides are transported basipetally together with sucrose via the phloem whereas tZR is transported acropetally via the transpiration stream in the xylem. Moreover, different import carriers are involved in cell-cell transport of free bases and cytokinin nucleosides (for a recent review [32]). The observed reduction in iPA, might indicate an impaired phloem derived transport of iPA towards the roots upon expression of *Spcdc25*. Hence, our current data highlight subtle cross talk between *Spcdc25 *expression and cytokinin supply.

Lateral root primordia start in pericycle cells opposite protoxylem poles. When secondary root founder cells are activated, cycling is started again in cells previously held in G2 [33,34]. This is under control of a complex interplay of auxin and cytokinins at the level of biosynthesis and transport. Spcdc25 replaces the normal cytokinin requirement in the CDK dephosphorylation during G2-M resulting in an active CDK in order to unblock the substrate and ATP-binding sites [14]. Notably, in animal cells, cytokinins act as a competitive inhibitor for ATP binding on CDC2 (iP, tZ, cZ), CDK5 (iP, tZ, cZ) and CDK4 (iP is exclusive inhibitor). Although CDK4 has no orthologue *in planta*, it is clear that iP is an exclusive cytokinin in the control of the G1/S transition in animal cells [35]. Hence, the reduction in iPA concentration in the root reported here may be important in cell cycle control in clusters of pericyle cells that serve as secondary root founders.

Another strong feature of enhanced root branching in Arabidopsis is positive regulation by IAA as demonstrated through both genetic and physiological manipulations [33,36,37]. Thus, the greater the amount of IAA relative to cytokinins in primary roots, the greater the frequency of lateral roots. Notably, we also detected an increased amount of IAA in roots of Spcdc25 compared with WT, which again is consistent with the strong rooting phenotype reported here.

### *Spcdc25 *expression alters root architecture

*Spcdc25 *had a null effect on primary root elongation yet its expression altered the architecture of the meristem and the overall width of the root. Notably, cells in the cortex and stele were smaller but more isodiametric in Spcdc25 compared with wild type, caused by a reduction in cell width but not cell length. Körper type "⊥" divisions occur in apical initials of RAMs whereby a periclinal division creates two files of cells that then form lineages as a result of repeated transverse divisions [38]. Hence, the periclinal division of apical initials has a profound effect on the overall width of the RAM. Here we hypothesise that premature entry into mitosis, activated by *Spcdc25 *expression, restricts the width of these periclinal divisions in both cortex and stele with the net result being a narrower RAM and a narrower primary root system compared with WT. Spcdc25 can indeed alter the plane of cell divisions to induce isodiametric growth of BY-2 cells and other tobacco lines in culture [17,39]. Note that in cultured tobacco cells, transverse divisions are the norm and that isodiametric cells come about through an increased frequency of longitudinal (periclinal) divisions. This type of effect is also suggested to result in an increased frequency of periclinal divisions in the pericycle producing the strong lateral root phenotype in both tobacco [15] and here in Arabidopsis.

### *Spcdc25 *expression perturbs ethylene signalling

An induction of isodiametric cell growth is a classic ethylene response [25] and the hypersensitive hypocotyl shortening response reported here either in the presence or absence of exogenous ethylene and its overproduction in Spcdc25 plants all point to an ethylene effect at the cellular level. To our knowledge, an ethylene induced isodiametric cell phenotype in RAMs has not been overtly reported in the published literature although in a study of ethylene effects on the quiescent centre of Arabidopsis, images of WT RAMs treated with ACC were narrower than untreated controls (Figure 3A, B and 3E of [40]). Nevertheless, the up-regulation of ethylene associated genes, hypocotyl length suppression in darkness see also, [41] and isodiametric RAM cellular phenotype tend to suggest that changes in ethylene synthesis or signalling in Spcdc25 plants are regulating these effects. Previous work established that exogenous ethylene has no substantial effect on rates of cell division in RAMs of either pea or corn [42] nor did we detect any difference in primary root elongation rates in Spcdc25 compared with WT. However, in Arabidopsis ethylene does modulate cell division in the quiescent centre [40] and through interactions with auxin assists in the regulation of root cap size [43].

## Conclusions

**Figure 6:**
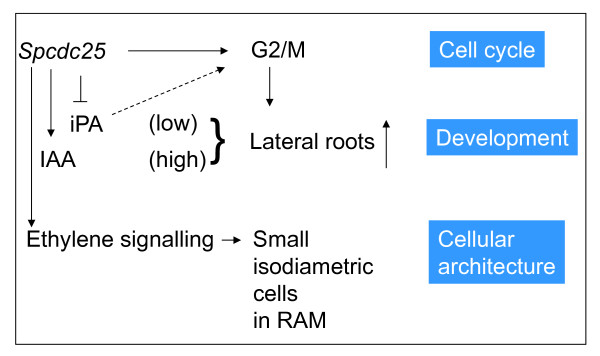
**Cross talk between *Spcdc25*, hormonal signalling and development**. *Spcdc25 *depletes and replaces cytokinins at the G2/M transition of cell cycle (Orchard et al., 2005), depletes iPA and raises IAA to promote lateral root formation and stimulates ethylene signalling to promote small isodiametric cells in cortex and stele of primary roots (data reported here)

In conclusion, we demonstrate that ectopic expression of the mitotic inducer gene, *Spcdc25 *in Arabidopsis has several important but explainable consequences. At a developmental level, *Spcdc25 *expression depletes iPA levels, down regulates *AHK3 *expression but raises IAA levels and promotes lateral root production. This alteration in the endogenous auxin-cyokinin milieu is entirely consistent with the stimulation of lateral roots. At a cellular level, *Spcdc25 *perturbs ethylene signalling, induces the plants to over produce ethylene that in turn probably alters planes of cell division that lead to isodiametric cell growth in RAMs. We have shown previously that *Spcdc25 *expression causes premature mitosis at a reduced cell size through shortening of the G2 phase of the cell cycle and bypasses a requirement for cytokinins at the G2/M transition [17]. Hence our data are important in highlighting cross talk between cell cycle control, hormonal signalling and development (Figure 6).

## Methods

### Transformation of Arabidopsis and plant growth

The *Spcdc25 *construct, BTX::Spcdc25, comprising the *Spcdc25 *ORF driven by an attenuated 35S CaMV promoter as described in [17], was used to transform *Arabidopsis thaliana cv*. Columbia plants by the floral dip method [44]. Expression of the transgene was checked in several independent transformant lines by RT-PCR using primers specific for the transgene P7 5'-TTAGGTCCCCTTCTCCGATG and P101 5'- TCAATGAGTCCTCCCTTCACG). Two lines were selected for their expression profile (BTX::Spcdc25 line 9 and line 10). Initial experiments showed that the phenotype of the two lines was very similar and hence further experiments were performed only with line 9. From here on, the genotypes used in this study are referred to as WT (wild type) or Spcdc25 (BTX::Spcdc25 expressing) plants.

For root measurements, seeds were surface sterilised and sown aseptically 1.5 cm apart onto Murashige and Skoog (MS) medium [45] agar in 90 mm diameter Petri-dishes. Seedlings were stratified at 6°C for 24 h and grown vertically at 21°C with 16 h light (fluence rate = 300 μm m^-2 ^s^-1^) 8 h darkness in a Sanyo-Gallenkamp Arabidopsis chamber. To score lateral root primordia, seedlings were fixed in 3 to 1 absolute ethanol to glacial acetic acid and Feulgen stained [46]. Primary root length, numbers of lateral roots and lateral root primordia were recorded using a stereo dissecting microscope (Nikon Z100). Additionally, hypocotyls were dissected from 24 day old seedlings and the number of adventitious roots were scored.

### Analysis of root apical meristem phenotypes

Ten day old seedlings grown as above were used for the RAM analyses. Roots were fixed and mounted on slides in 8:3:1 chloral hydrate:distilled water: glycerol taking care to apply a coverslip gently [47]. Cell length, width and number were measured in three tissues of the RAM: epidermis, cortex and stele using a ZeissAxiophot set for D.I.C. interfaced to Image analysis software (PixiLINK (C) Capture S.E.). Measurements were taken along longitudinal cell files of epidermis, cortex and mid-stele until the parameter spanned a cell that suddenly increased its cell length/width substantially compared with the previous one. For all genotypes, and for all tissues, this was between a 1.40- to 1.95-fold increase. This was the transition point beyond which those cells began to elongate substantially and is taken to be the basipetal border of the promeristem for each tissue. The cellular measurements were undertaken at either x20 or x40. Images of whole root tips were captured using a low power, x10 objective.

For confocal images, roots were prepared as described in [48] and visualised using a Leica TCS SP2 (Spectral Confocal and Multiphoton System) confocal scanning laser microscope interfaced to ImageJ software (ImageJ64).

### RNA extraction and real time RT-PCR

RNA extractions from roots of Spcdc25 and WT seedlings, grown as described above, were performed using 2 ml TRI reagent (SIGMA-ALDRICH, Dorset, UK) according to the manufacturer's protocol, following grinding to a powder in liquid nitrogen using a mortar and pestle. RNA was treated with RQ1 Dnase (PROMEGA, Southampton, UK) to remove residual genomic DNA. cDNA was synthesised as described in [49].

Seedling root cDNA (30 ng) was amplified in qRT-PCR reactions in a 20 μl total volume containing: 10 μl 2× PowerSYBR Green PCR Master Mix (Applied Biosystems); 400 nM of each primer; 30 ng cDNA. Reactions were performed in triplicate with the following cycles: 95°C for 10 min, 40 cycles of 95°C for 15 s and 60°C for 1 min. To test primer specificity, melting curve analysis (from 60°C to 95°C with an increasing heat rate of 0.5°C s^-1^) was performed after amplifications.

Relative quantification of gene expression data was carried out with the 2^-DDCT ^or comparative CT method [50]. Expression levels were normalized with the CT values obtained for the housekeeping gene *UBQ10 *(At4G05320F, 5'- CACACTCCACTTGGTCTTGCGT-3'; At4G05320R, 5'- TGGTCTTTCCGGTGAGAGTCTTCA-3'; [51]. The primers used for qRT-PCR of target genes *ERF/AP2 *(At5g61590), *ACC oxidase *(At1g12010), *EBF1 *(At2g25490) and *AHK3 *(At1g27320) are listed in Additional file 6.

### Microarray analysis

Messenger RNA was amplified from purified total RNA using the MessageAmp aRNA kit (AMBION, Huntingdon, Cambs, UK) according to the manufacturer's protocol from 5 μg extracted total RNA. The CyScribe Post-Labelling Kit (AMERSHAM BIOSCIENCES, Little Chalfont, Bucks. UK) was used to label 1 μg aRNA, with either Cy3 or Cy5 according to the manufacturers' instructions, using 1 μg of aRNA. Each labelled RNA pair was freeze-dried and resuspended in 50 μl of hybridisation buffer containing 25% formamide, 5 × SSC, 0.1% SDS, 0.5 mg/ml poly(dA), 0.5 mg/ml yeast tRNA. CATMA v 3 arrays comprising 24 000 gene probes ([52]; http://www.catma.org) were hybridised with the labelled RNA which was heated to 95°C for 5 min and applied to the microarray slide surface. A second microarray slide was lowered over the labelled RNA and the slides were hybridised back-to-back overnight in a humid chamber at 42°C. Four slides were used for hybridisations including a dye swap for each probe pair. Following hybridisation, the slides were washed and scanned using an Affymetrix 428 array scanner with the supplied software (AFFYMETRIX, Santa Clara, CA, USA) at 532 nm (Cy3) and 633 nm (Cy5). Scanned images were quantified using Imagene version 5 software (BIODISCOVERY, El Segundo, CA, USA). Spot quality labelling (flags) was defined for empty spots with a signal strength threshold of 1, and for shape regularity with a threshold of 0.4. The median signal intensity across each spot and the median background intensity were calculated in both channels, and these data were exported into GeneSpring version 6 (AGILENT, Santa Clara, CA, USA). Background intensity was subtracted from spot intensity for both channels giving the background-corrected spot intensity.

Data analysis was carried out using the GeneSpring microarray data analysis package. Ratios between mutant and wild type expression were calculated and levels for the four replicates were averaged. Genes showing an average ratio above 2 or below 0.5 were selected and, following a Benjamini and Hochberg false discovery rate test (BHFDR), genes with P value > 0.05 were excluded.

### Hypocotyl and ethylene measurements

WT and Spcdc25 seeds were surface sterilised and stratified as above and seedlings grown in either continuous dark or 16 h L/8 h D at 21°C. Hypocotyl length was recorded at 3, 4 and 7 days following germination. Effects of exogenous ethylene were measured by sealing plates with Nescofilm and injecting 0, 10 or 100 ppm ethylene through a predrilled, sealed hole in the lid of the Petri dish. Hypocotyl measurements were then made as above. To measure endogenous ethylene, 0.015 g seeds of WT or Spcdc25 were sown onto the surface of 5 ml 1 × MS in 10 ml glass head space vials (Chromacol, Thermo Scientific, Loughborough, UK), seeds were stratified at 4°C for 24 h then grown at 21°C for 10 days in the dark. Headspace concentration of ethylene was measured on a Clarus 500, modified model 2101 analyzer (PerkinElmer, MA, USA), retention time was confirmed using pure ethylene and quantification was calibrated using a standard gas mixture (Scott Specialty Gases, mixture 54).

### Cytokinin and IAA measurements

Frozen samples were ground in liquid nitrogen and transferred to Bieleski [53] solvent (10 ml/g fresh weight). Deuterated standards (100 pmol [^13 ^C_6_-phenyl]- IAA (Cambridge Isotope Laboratory Inc., Andover, MA, USA) and 10 pmol each for cytokinins ([^2^H_5_]DHZ, [^2^H_5_][9R]DHZ, [^2^H_5_](9 G)DHZ, [^2^H_6_]iP, [^2^H_6_][9R]iP, [^2^H_6_](9 G)iP, [^2^H_5_](7 G)DHZ, [^2^H_5_](OG)DHZ, [^2^H_5_](OG)[9R]DHZ, [^2^H_6_](7 G)iP), OlchemIm Inc. Olomouc, Czech Rep) were added to the extracts and incubated overnight at -20°C. Cytokinins were purified by solid phase extraction combining DEAE-Sephadex (HG Healthcare, Uppsala Sweden) and RP-C18 (500 mg, Varian, Middelburg, The Netherlands) cartridges. Purified extracts were loaded onto immunoaffinity columns containing monoclonal antibodies against isoprenoid cytokinins (OlchemIm). Cytokinins were analysed by UPLC-TQD (ACQUITY TQD mass spectrometer (Waters, Milford, MA, USA) using an ES + interphase (based on [54,55]). Chromatograms were analysed using Masslynx and Quanlynx v4.1 software (Waters, Milford, MA, USA) and cytokinin concentrations were calculated according to the principles of isotope dilution.

## Abbreviations

CDK: Cyclin-dependent kinases; Kin: Kinetin; NAA: Naphthyl acetic acid; Tyr15: Tyrosine15.

## Authors' contributions

Natasha Spadafora undertook expression analyses and real time PCR, root phenotyping, tissue culture grids, confocal microscopy and hypocotyl growth analyses, David Parfitt undertook microarray analyses, Angela Marchbank and Li Sherong created transgenic plants, Leonardo Bruno assisted with real time PCR and confocal microscopy, Rhys Vaughan undertook measurements of ethylene sensitivity and ethylene production; Jeroen Nieuwland assisted with DIC microscopy; Vicky Buchanan-Wollaston assisted with microarray analyses, Robert Herbert assisted with expression analyses and ethylene measurements, Beatrice Bitonti assisted with real time PCR and the writing of the manuscript, John Doonan assisted with expression analyses, Diego Albani assisted with DIC microscopy, Els Prinsen undertook endogenous hormone measurements, Dennis Francis assisted with phenotype analyses, DIC microscopy and drafted the manuscript. Hilary Rogers assisted in expression analyses, ethylene measurements and jointly with DF drafted the final version of the manuscript. All authors contributed at the draft stage of writing and all approved the final manuscript.

## Supplementary Material

Additional file 1 **Expression levels of *Spcdc25 *in transgenic lines of Arabidopsis**. RT-PCR of BTX::Spcdc25 lines 9 and 10 showing expression of the transgene in both lines (lower band is primer dimer). Click here for file

Additional file 2 **Figure S2**. Null effect of *Spcdc25 *expression on cell length in mature regions of primary root. DIC/Nomarski images of regions of primary roots exhibiting young lateral root primordia (YLRP) in WT and Spcdc25. Mean (± SE) cortical cell length. WT = 75.14 ± 10.10 Spcdc25 = 56.86 ± 11.61 μm. n = 25. Bar scale = 40 μm. Click here for file

Additional file 3 **Expression of *Spcdc25 *induces increased rooting in cultured hypocotyls at NAA/Kin: 50/100, 50/200, 25/50, 25/200**. Hypocotyls of 14 d old plants of (A) wild type (WT) (B) BTX::*Spcdc25 *(line 9) cultured on two-way concentration gradients of increasing concentrations (ng ml^-1^) of naphthyl acetic acid (NAA) and kinetin (Kin) for 30 d; representative grids from 3-6 replicate experiments. Cells boxed indicate the NAA/Kin concentrations used for further analysis. Scale bar = 1 mm. Click here for file

Additional file 4 **Table showing genes up-or down-regulated by 2-fold or more (Spcdc25/WT)**. Click here for file

Additional file 5 **Real time PCR verification of the microarray results**. Above each histogram bar (Spcdc25/WT) is the microarray result (Spcdc25/WT). n = 3. Click here for file

Additional file 6 **Table of real time PCR Primers**. Click here for file
