# Supplementary material for: Perturbation of cytokinin and ethylene-signalling pathways explain the strong rooting phenotype exhibited by Arabidopsis expressing the Schizosaccharomyces pombe mitotic inducer, cdc25
Source: BMC Plant Biol. 2012 Mar 27;12:45. doi: 10.1186/1471-2229-12-45 (PMC3362767; doi:10.1186/1471-2229-12-45)
Supplement: Additional file 1 — Expression levels of Spcdc25 in transgenic lines of Arabidopsis. RT-PCR of BTX::Spcdc25 lines 9 and 10 showing expression of the transgene in both lines (lower band is primer dimer). [file 1471-2229-12-45-S1.PPT]

## Slide 1
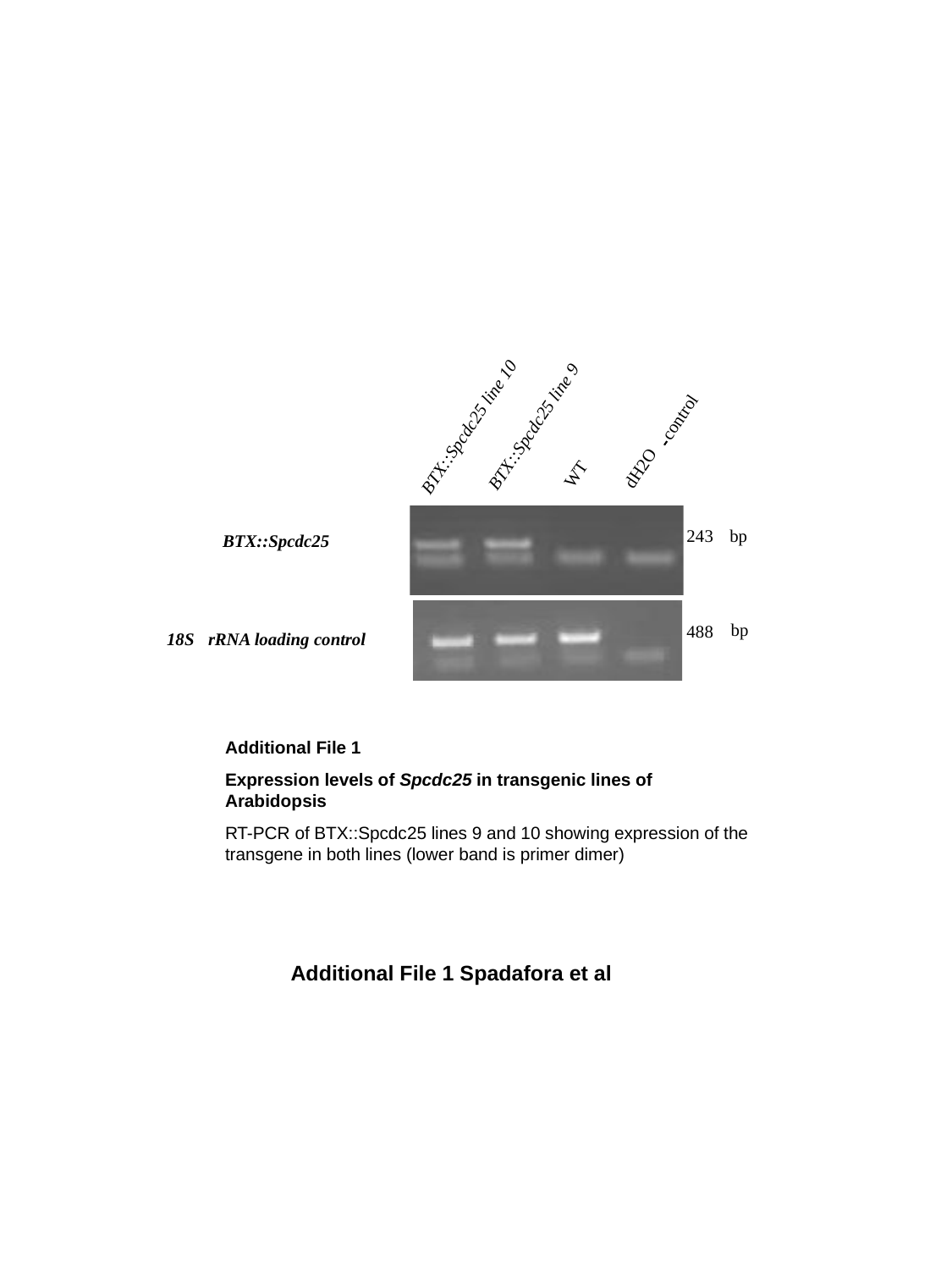

10
9
control
control
BTX::Spcdc25 line 9
BTX::Spcdc25 line 10
-
-
-
-
-
-
BHTXSpcdc25
BHTXSpcdc25
dH2O
dH2O
WT
WT
243
bp
BTX::Spcdc25
bp
488
18S
rRNA loading control
Additional File 1
Expression levels of Spcdc25 in transgenic lines of Arabidopsis
RT-PCR of BTX::Spcdc25 lines 9 and 10 showing expression of the transgene in both lines (lower band is primer dimer)
Additional File 1 Spadafora et al
