# Supplementary material for: Perturbation of cytokinin and ethylene-signalling pathways explain the strong rooting phenotype exhibited by Arabidopsis expressing the Schizosaccharomyces pombe mitotic inducer, cdc25
Source: BMC Plant Biol. 2012 Mar 27;12:45. doi: 10.1186/1471-2229-12-45 (PMC3362767; doi:10.1186/1471-2229-12-45)
Supplement: Additional file 2 — Figure S2. Null effect of Spcdc25 expression on cell length in mature regions of primary root. DIC/Nomarski images of regions of primary roots exhibiting young lateral root primordia (YLRP) in WT and Spcdc25. Mean (± SE) cortical cell length. WT = 75.14 ± 10.10 Spcdc25 = 56.86 ± 11.61 μm. n = 25. Bar scale = 40 μm. [file 1471-2229-12-45-S2.PPT]

## Slide 1
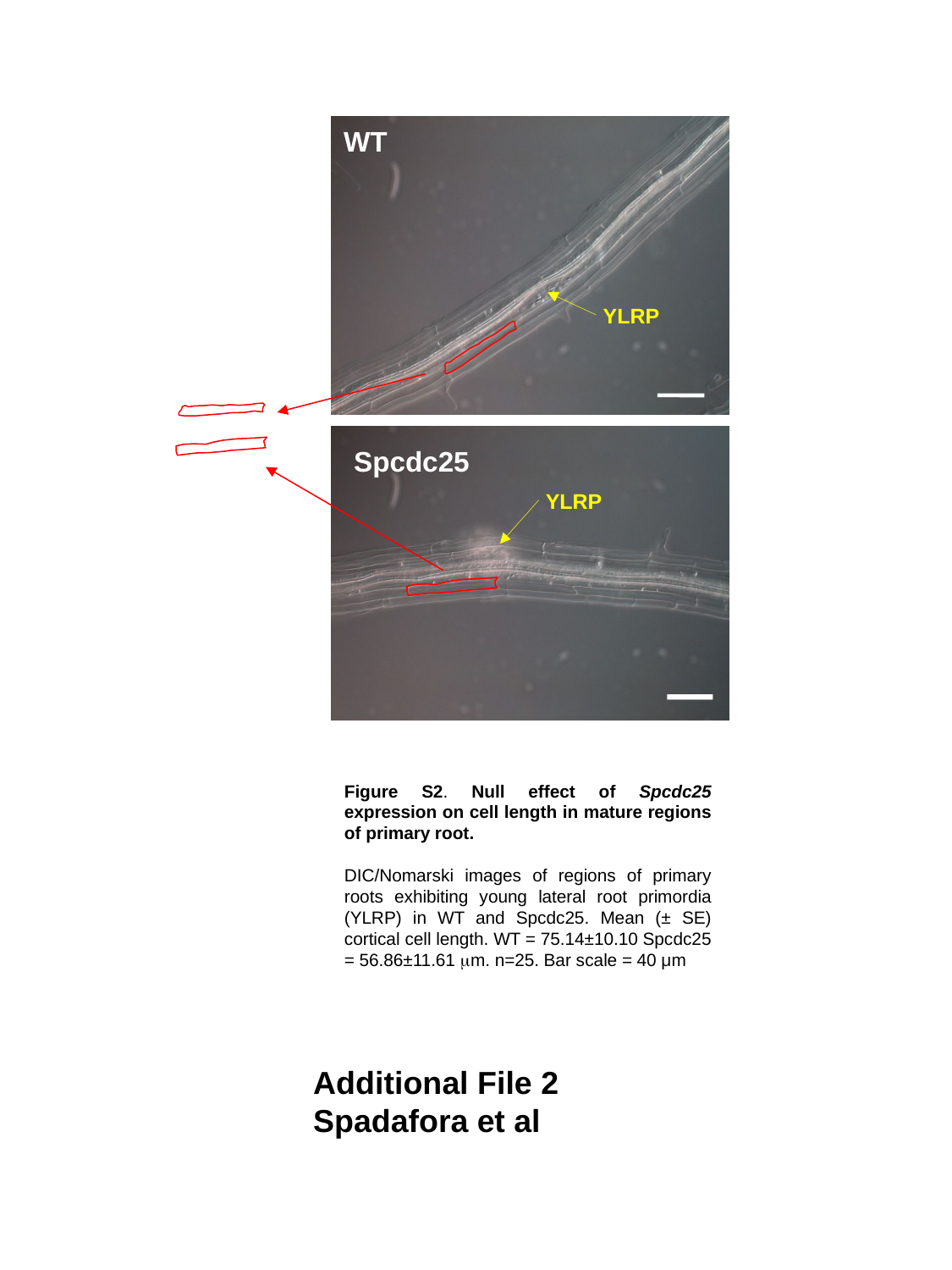

WT
WT
YLRP
Spcdc25
YLRP
Figure S2. Null effect of Spcdc25 expression on cell length in mature regions of primary root.
DIC/Nomarski images of regions of primary roots exhibiting young lateral root primordia (YLRP) in WT and Spcdc25. Mean (± SE) cortical cell length. WT = 75.14±10.10 Spcdc25 = 56.86±11.61 μm. n=25. Bar scale = 40 μm
Additional File 2 Spadafora et al
