# Supplementary material for: Perturbation of cytokinin and ethylene-signalling pathways explain the strong rooting phenotype exhibited by Arabidopsis expressing the Schizosaccharomyces pombe mitotic inducer, cdc25
Source: BMC Plant Biol. 2012 Mar 27;12:45. doi: 10.1186/1471-2229-12-45 (PMC3362767; doi:10.1186/1471-2229-12-45)
Supplement: Additional file 3 — Expression of Spcdc25 induces increased rooting in cultured hypocotyls at NAA/Kin: 50/100, 50/200, 25/50, 25/200. Hypocotyls of 14 d old plants of (A) wild type (WT) (B) BTX::Spcdc25 (line 9) cultured on two-way concentration gradients of increasing concentrations (ng ml-1) of naphthyl acetic acid (NAA) and kinetin (Kin) for 30 d; representative grids from 3-6 replicate experiments. Cells boxed indicate the NAA/Kin concentrations used for further analysis. Scale bar = 1 mm. [file 1471-2229-12-45-S3.PPT]

## Slide 1
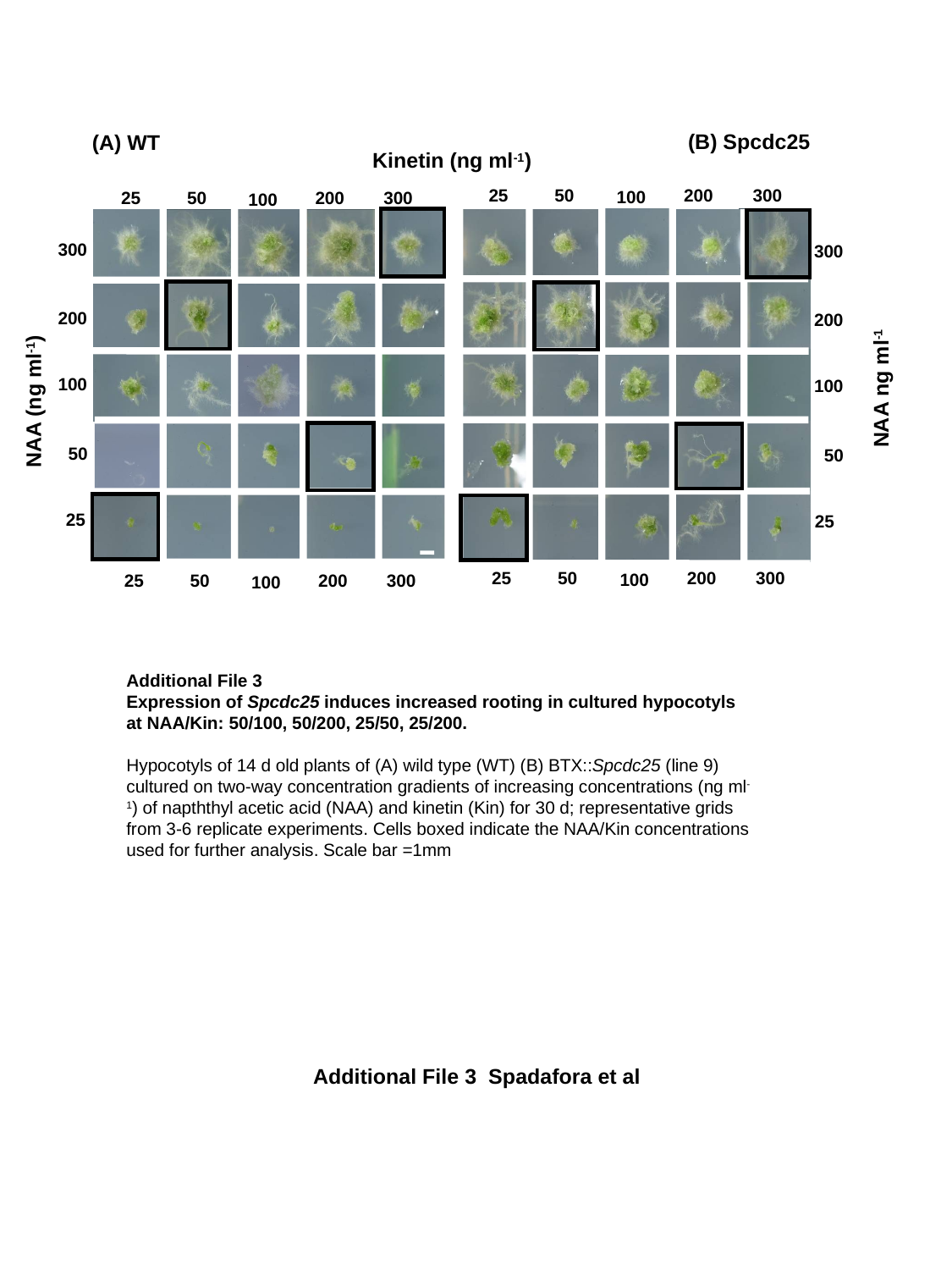

(B) Spcdc25
(A) WT
Kinetin (ng ml-1)
25
50
200
300
100
25
50
200
300
100
300
300
200
200
NAA ng ml-1
100
100
NAA (ng ml-1)
50
50
25
25
25
50
200
300
100
25
50
200
300
100
Additional File 3
Expression of Spcdc25 induces increased rooting in cultured hypocotyls at NAA/Kin: 50/100, 50/200, 25/50, 25/200.
Hypocotyls of 14 d old plants of (A) wild type (WT) (B) BTX::Spcdc25 (line 9) cultured on two-way concentration gradients of increasing concentrations (ng ml-1) of napththyl acetic acid (NAA) and kinetin (Kin) for 30 d; representative grids from 3-6 replicate experiments. Cells boxed indicate the NAA/Kin concentrations used for further analysis. Scale bar =1mm
Additional File 3 Spadafora et al
