# Supplementary material for: Perturbation of cytokinin and ethylene-signalling pathways explain the strong rooting phenotype exhibited by Arabidopsis expressing the Schizosaccharomyces pombe mitotic inducer, cdc25
Source: BMC Plant Biol. 2012 Mar 27;12:45. doi: 10.1186/1471-2229-12-45 (PMC3362767; doi:10.1186/1471-2229-12-45)
Supplement: Additional file 5 — Real time PCR verification of the microarray results. Above each histogram bar (Spcdc25/WT) is the microarray result (Spcdc25/WT). n = 3. [file 1471-2229-12-45-S5.PPT]

## Slide 1
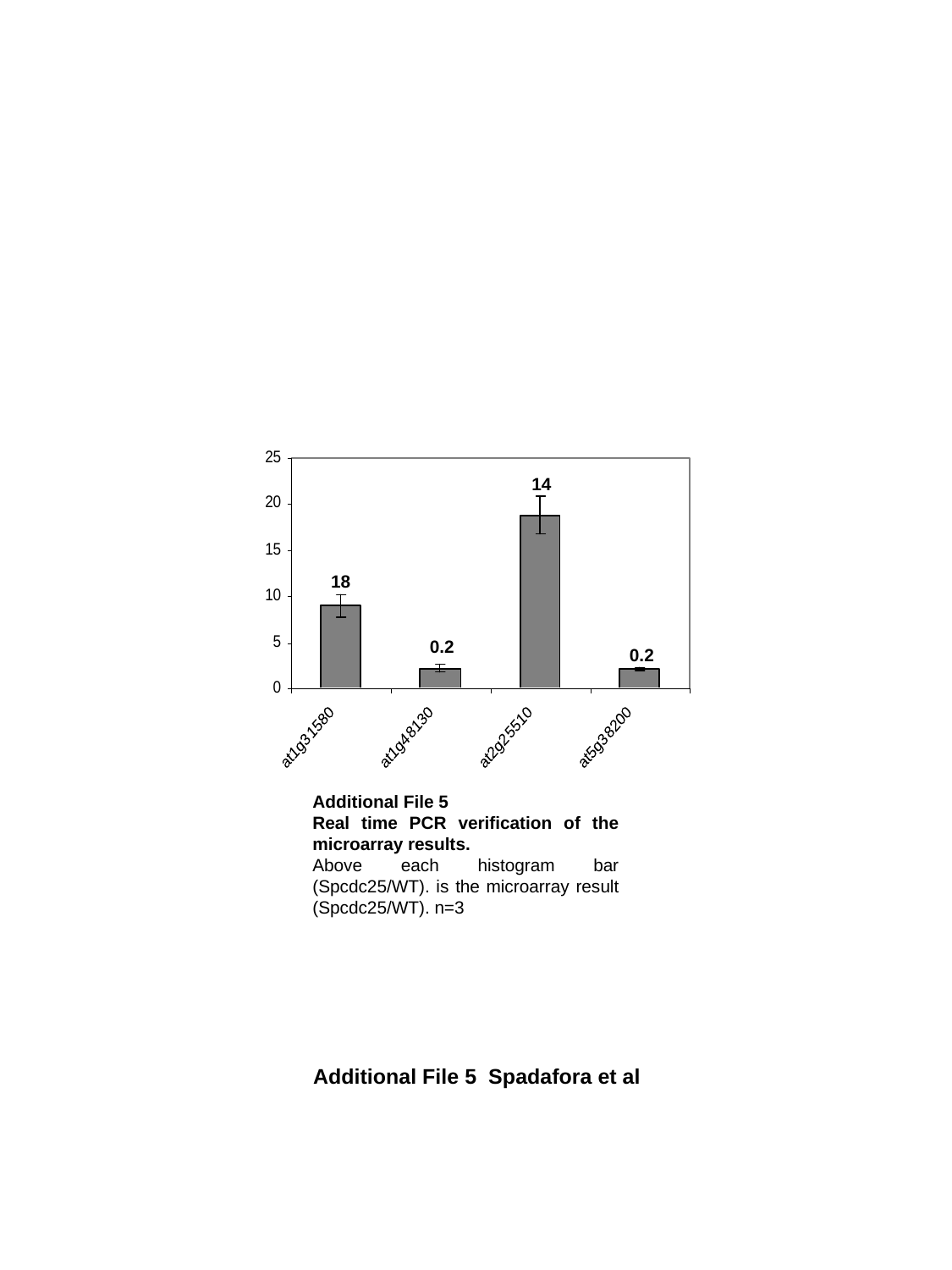

14
18
0.2
0.2
Additional File 5
Real time PCR verification of the microarray results.
Above each histogram bar (Spcdc25/WT). is the microarray result (Spcdc25/WT). n=3
Additional File 5 Spadafora et al
