# Supplementary material for: Perturbation of cytokinin and ethylene-signalling pathways explain the strong rooting phenotype exhibited by Arabidopsis expressing the Schizosaccharomyces pombe mitotic inducer, cdc25
Source: BMC Plant Biol. 2012 Mar 27;12:45. doi: 10.1186/1471-2229-12-45 (PMC3362767; doi:10.1186/1471-2229-12-45)
Supplement: Additional file 6 — Table of real time PCR Primers. [file 1471-2229-12-45-S6.DOC]

**Additional File 6**…:Real time PCR Primers

| Target gene | Primer name | Sequence 5’-3’ | Product size (bp) |
| --- | --- | --- | --- |
| At5g61590 *ERF/AP2* | At5g61590F  At5g61590R | GAAAGTATGATGCTCCGGTCAAT  GGCTGTGGTACATCGGTTCTC | 61 |
| At1g12010 | At1g12010F | GATGTGGGATCTTCTACGAAAGCT | 83 |
| *ACC oxidase* | At1g12010R | CATCAACCCCAATCCTTTTATT |  |
| At2g25490  *EBF1* | At2g25490F  At2g25490R | GATGGGAAGAAGGCAACAGATG  CCACGACCAGCAGTTCCAA | 62 |
| At1g27320  *AHK3* | At1g27320F  At1g27320R | CCAGCTGAAAGAAAGAATGACAAC  TCATGCCCCGGAATTCC | 58 |
